# Emerging Urinary Markers of Renal Injury in Obstructive Nephropathy

**DOI:** 10.1155/2014/303298

**Published:** 2014-07-02

**Authors:** Giuseppe Lucarelli, Vito Mancini, Vanessa Galleggiante, Monica Rutigliano, Antonio Vavallo, Michele Battaglia, Pasquale Ditonno

**Affiliations:** Department of Emergency and Organ Transplantation, Urology, Andrology and Kidney Transplantation Unit, University of Bari, 70124 Bari, Italy

## Abstract

The effects of obstruction on renal function are the consequence of many factors that profoundly alter all components of glomerular function. Besides the acute effects on glomerular filtration rate and tubule function, a chronic obstruction induces tubular and interstitial injury that results from the activation of different pathways. The progression of tubulointerstitial injury leads to chronic renal damage characterized by tubular atrophy, inflammatory cell infiltration, and interstitial fibrosis. Obstructive nephropathy is an evolving disease in which the renal damage continues even after relief of the obstruction. In particular, it has been demonstrated that the time of relief is the most important factor in predicting long-term renal function deterioration. In this setting, the EGF/MCP-1 ratio, urinary NGAL, and urinary KIM-1 are useful early biomarkers of progressive renal damage and could have a potential role in predicting the long-term renal outcome. This minireview summarizes the role of these emerging urinary biomarkers of obstructive nephropathy based on the current understanding of the pathophysiology of renal injury.

## 1. Background

The study as well as identification of potential biomarkers for obstructive nephropathy requires an in-depth understanding of the biological pathways involved in the pathogenesis of this disorder. The effects of obstruction on renal function are the consequence of many factors that profoundly alter all components of glomerular function. Besides the acute effects on glomerular filtration rate and tubule function, a chronic unilateral ureteral obstruction (UUO) induces tubular and interstitial injury that results from the activation of different pathways. The progression of tubulointerstitial injury leads to chronic renal damage characterized by tubular atrophy, inflammatory cell infiltration, and interstitial fibrosis [1].

It has been shown that renal tubular injury is the consequence of mechanical stretching, hypoxia, and exposure to oxygen free radicals that result from increased hydrostatic pressure, reduced blood flow, and increased oxidative stress.

**Figure 1:**
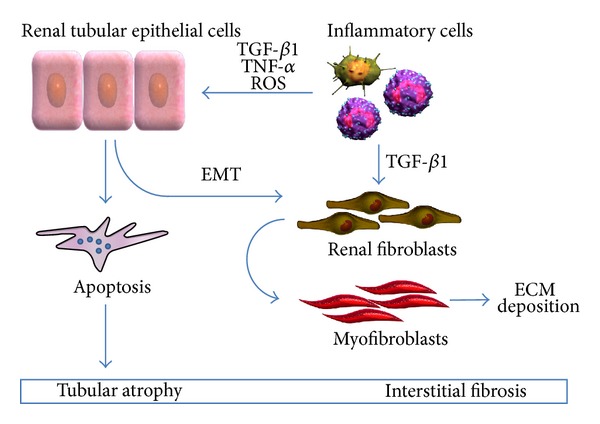
Renal cell types involved in the pathogenesis and progression of obstructive nephropathy. ECM: extracellular matrix; EMT: epithelial to mesenchymal transition; TGF-*β*1: transforming growth factor-*β*1; TNF-*α*: tumor necrosis factor-*α*; ROS: reactive oxygen species.

In this scenario, three cell types play a fundamental role in the pathogenesis and progression of renal damage: tubular epithelial cells, infiltrating inflammatory cells, and interstitial fibroblasts (Figure 1). During UUO, changes in both the frequency and amplitude of pyeloureteral contractions, in conjunction with sustained urinary pooling proximal to the site of obstruction, contribute to mechanical stretch injury of the tubular epithelium. It has been shown that mechanical stretching of tubular cells is transduced via the extracellular matrix- (ECM-) integrin-cytoskeleton complex [2, 3]. Moreover, recent studies have described the transient receptor potential cationic channel-1 (TRPC-1) as a potential stretch-activated calcium channel expressed in renal cells [4]. Modifications in the intratubular dynamic force as a consequence of obstruction can induce the upregulation and release of transforming growth factor-*β* (TGF-*β*) and the activation of tubular apoptosis and the nuclear factor-kB (NF-kB) pathway [2, 5].

In the past year, the deregulation of many cell signaling pathways has been demonstrated in obstructive nephropathy. Among these, the intrarenal renin-angiotensin system plays a fundamental role in orchestrating all the elements that are hallmarks of renal damage. Angiotensin II (ANG II), in particular, can upregulate the expression of many factors such as transforming growth factor-*β*1 (TGF-*β*1), tumor necrosis factor-*α*, (TNF-*α*), osteopontin, vascular cell adhesion molecule-1 (VCAM-1), and NF-kB [6, 7]. ANG II increases the expression of some proliferative factors, including platelet-derived growth factor (PDGF) and basic fibroblast growth factor (bFGF). ANG II also stimulates oxidative stress, through NADPH oxidase activity and the production of reactive oxygen species (ROS) and by increasing NO catabolism. Experimental studies have indicated that protection from obstruction-induced renal damage could be achieved by NO supplementation. This can be accomplished either by inhibiting angiotensin converting enzyme, which increases NO formation, or by stimulating endogenous NO synthase (NOS) [8, 9]. Chronic UUO in mice leads to a significant reduction in inducible NOS (iNOS) activity, and the obstructed kidneys of iNOS knockout mice showed a higher number of apoptotic renal tubules than wild type controls [10].

**Figure 2:**
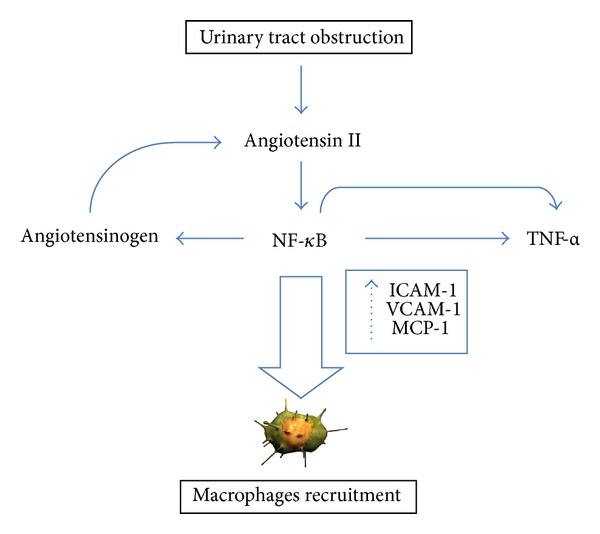
Autocrine-reinforcing loops amplifying angiotensin II (ANG II) and tumor necrosis factor-*α* (TNF-*α*) signalling. NF-kB: nuclear factor kappa-light-chain-enhancer of activated B cells; ICAM-1: intercellular adhesion molecule-1; MCP-1: monocyte chemotactic protein-1; VCAM-1: vascular cell adhesion molecule-1.

It has also been demonstrated that the angiotensinogen gene, coding for the precursor of angiotensin, is stimulated by NF-kB activation and an autocrine-reinforcing loop between NF-kB and TNF-*α* has been described [11, 12]. According to this model, ANG activation II stimulates NF-kB production, which in turn fuels at least two autocrine-reinforcing loops that amplify ANG II and TNF-*α* formation (Figure 2).

All these factors, along with the upregulation of adhesion molecules such as VCAM-1 and ICAM-1, lead to the recruitment of inflammatory cells within interstitial spaces. These cells, in turn, release additional cytokines with profibrotic and proapoptotic activities, amplifying the tubular damage.

**Figure 3:**
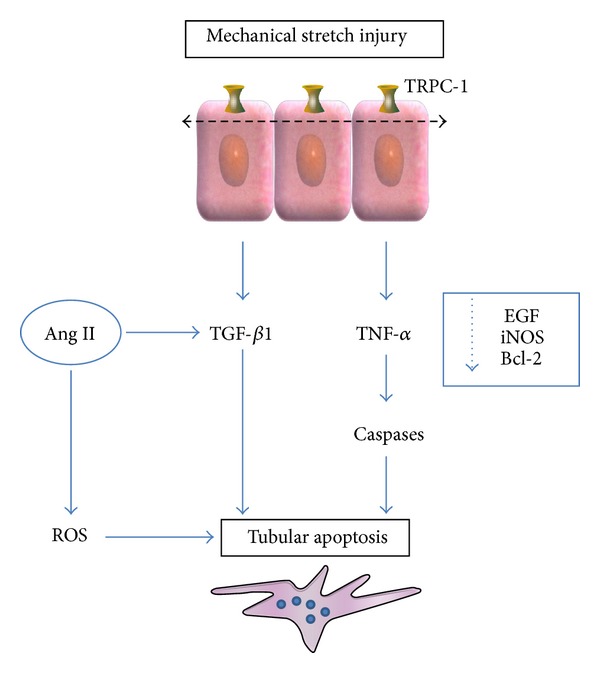
Pathogenesis of renal tubular apoptosis in obstructive nephropathy. Ang II: angiotensin II; EGF: epidermal growth factor; iNOS: inducible NO synthase; ROS: reactive oxygen species; TRPC-1: transient receptor potential cationic channel-1.

Apoptosis of tubular and interstitial cells is also presumed to be the cause of tubulointerstitial atrophy, secondary to obstructive nephropathy. Apoptosis may be activated by a large number of factors, several of which are present in obstructive nephropathy, such as ischemia, hypoxia, growth factors, cytokines, ANG II, TNF-*α*, reactive oxygen species, and mechanical stretching. These factors act on a family of cell membrane receptors that include the TNF receptor and Fas. The end result of the activation of these receptors is mitochondrial destabilization and the release of cytochrome C, which subsequently triggers the caspase-mediated apoptotic pathway. The release of cytochrome C is also promoted by the downregulation of antiapoptotic protein bcl-2 [13, 14] (Figure 3).

The activation of all these processes has, as the final common pathway, the development of interstitial fibrosis due to increased deposition of ECM, cell infiltration, tubular apoptosis, and induction of the epithelial to mesenchymal transition (EMT). This latter process involves the tubular cells and is characterized by the downregulation of epithelial markers (such as E-cadherin, ZO-1, and cytokeratins), upregulation of mesenchymal proteins (including vimentin, *α*-smooth muscle actin, and FSP-1), loss of cell adhesion molecules, invasion of basement membrane, and migration in the interstitium, where these cells acquire myofibroblast characteristics [15]. In this compartment myofibroblasts induce collagen accumulation and are the main ECM-producing cells. Renal fibrosis is also induced by different cytokines and growth factors. Among these, TGF-*β*1 is the most powerful profibrogenic factor involved in kidney diseases and the major mediator of renal injury during UUO.

In this minireview we summarize the role of the emerging urinary biomarkers of obstructive nephropathy based on the current understanding of the pathophysiology of renal injury.

## 2. Urinary Epidermal Growth Factor (EGF), Monocyte Chemotactic Protein-1 (MCP-1), and EGF/MCP-1 Ratio

Many studies have explored the molecular events associated with the development of tubular atrophy and interstitial fibrosis induced by chronic urinary tract obstruction. Notably, ureteral obstruction determines a significant increase in monocyte chemotactic protein-1 (MCP-1) expression and a decrease in epidermal growth factor (EGF) expression by tubular cells [7–10]. MCP-1 is a specific chemokine that promotes monocyte chemotaxis, and its expression at the tubular level drives the recruitment of these inflammatory cells within the interstitial space of the obstructed kidney. An increased expression of this protein has been observed in different tubulointerstitial diseases [16–19].

MCP-1 renal expression and urine excretion are strictly related to tubular damage and the extent of monocyte infiltration. As described above, in an obstructed kidney, tubular epithelial cells release a number of autocrine factors and cytokines, including ANG II, TGF-*β*1, and TNF-*α* [20–24]. These factors, along with overexpression of adhesion molecules, lead to infiltration of the renal interstitium by inflammatory cells, including macrophages. These, in turn, release additional cytokines.

All of these factors accelerate the development of interstitial fibrosis by increasing the extracellular matrix, epithelial to mesenchymal transition, cell infiltration, and tubular apoptosis. On the other hand, EGF, a polypeptide produced by the ascending portion of Henle's loop and by the distal convoluted tubule, modulates tubular cell growth and tissue response to injury in the kidney with tubulointerstitial damage [25–28]. In obstructive nephropathy a downregulation of EGF, Bcl-2, and antioxidant enzymes has been observed in association with an increased production of superoxide and hydrogen peroxide, contributing to an increased rate of apoptosis and tubular dropout. EGF administration reduces UUO-induced renal damage by increasing tubular proliferation and reducing apoptosis, tubular atrophy, and interstitial fibrosis. In view of these findings, a reduced urinary EGF/MCP-1 ratio has been proposed as a marker of acute and chronic damage in human renal diseases [16, 17, 29].

The deregulation of these molecular pathways is associated with tubulointerstitial fibrosis and permanent loss of renal function, which may continue to progress even after the obstruction has been relieved. In a neonatal rat model, Chevalier et al. showed the progression of renal interstitial collagen accumulation after relief of UUO [30]. Moreover, in another study, the same authors demonstrated that despite relief of five-day obstruction in the neonatal period, the growth of the kidney in adulthood was impaired. In particular a significant reduction of nephron number in the postobstructed kidney was observed, in association with an increased expression of interstitial *α*-smooth muscle actin and macrophage infiltration [31]. Ito et al. demonstrated, in adult rats, that even if the renal blood flow and GFR of an obstructed kidney returned to control levels after relief of a short-term ureteral obstruction, in the long term renal function was compromised by progressive interstitial fibrosis and tubular atrophy [32]. It is well known that the recovery of renal function after relief of ureteral obstruction depends on several factors including the location and duration of the obstruction, whether it is complete or partial, and the presence of infection. In particular, time before relief seems to be the most critical issue. A recent study showed that patients who underwent delayed relief of a ureteral obstruction had a decreased long-term renal function and were at risk for arterial hypertension [33]. Moreover, the median urinary EGF/MCP1 ratio was significantly higher in the subgroup of patients who underwent repair of the ureteral lesion within 2 weeks compared to those with a later repair. A direct correlation was found between MAG3 clearance of the obstructed unit and the EGF/MCP-1 ratio, as well as an inverse correlation between the urinary cytokines ratio and time before repair. The role of the EGF/MCP-1 ratio as a marker of renal damage has also been explored in children with congenital obstructive nephropathy. Grandaliano et al. observed a significant reduction of EGF urinary excretion in subjects affected by congenital ureteropelvic junction obstruction (UPJO) compared to healthy children, in association with a marked increase of MCP-1 levels [16]. Moreover, the MCP-1 urine concentration was significantly higher in patients with recurrent urinary infections. These findings are in agreement with other studies showing the same pattern of gene expression at renal tubulointerstitial level for these molecular markers [34–36]. A recent study confirmed the diagnostic role of the urinary EGF/MCP-1 ratio in a paediatric population affected by UPJO and suggested that these biomarkers may help to follow the progression of parenchymal damage in obstructed renal units [17].

## 3. Neutrophil Gelatinase-Associated Lipocalin (NGAL)

Neutrophil gelatinase-associated lipocalin (NGAL) is a 25 kDa protein of the lipocalin family [37]. It is not an organ-specific protein and is secreted by different tissues, including the respiratory, gastrointestinal, and urinary tracts. NGAL overexpression has been described in different pathological conditions such as inflammation, sepsis, ischemia, renal damage, and cancer. In the kidney, this protein is synthesized in the thick ascending limb of Henle's loop and collecting ducts [38, 39]. The expression of this protein is rapidly induced in response to renal tubular injury, and increased levels of serum and urinary NGAL (uNGAL) have been reported in the setting of different renal diseases such as acute kidney injury (AKI), diabetic nephropathy, nephritic syndrome, tubulointerstitial damage, and IgA nephropathy [40–43].

The induction of NGAL serves to limit tubular injury, even apart from its bacteriostatic properties. Although NGAL is synthesized in the distal nephron, it has been suggested that NGAL could act on the proximal nephron through its uptake from the circulation by tubular epithelia via endocytosis. According to this two-compartment model, in the setting of sepsis or renal disease, urinary NGAL is produced by local distal tubule synthesis, whereas proximal tubule NGAL derives from the circulating pool [39].

The role of NGAL as a biomarker of AKI was recently reviewed by Haase-Fielitz et al. [44]. In their meta-analysis, the authors found that serum or uNGAL levels represented a valuable early predictor of renal damage and that high concentrations had a prognostic role in predicting the progression to renal replacement therapy and mortality. A recent case-control prospective study evaluated the role of urinary NGAL in a population of children with severe hydronephrosis caused by UPJO [45]. The findings of this study showed that the uNGAL/creatinine ratio was significantly higher in obstructed patients compared to normal subjects. Moreover, three months after surgery, uNGAL values had decreased and did not significantly differ from the control group. Similar findings have been confirmed by more recent studies. In particular, Cost et al. showed, in a cohort of children with UPJO, that renal pelvis uNGAL levels were higher than bladder uNGAL and that these values were significantly higher compared to the levels measured in a control population of unaffected children [46]. Moreover, the renal pelvic uNGAL levels were inversely correlated with the function of the affected renal unit. The authors concluded by suggesting the potential usefulness of this biomarker for selecting patients at risk for renal function deterioration and candidates for reconstructive surgery.

## 4. Kidney Injury Molecule-1 (KIM-1)

Kidney injury molecule-1 (KIM-1) is a member of the type I transmembrane glycoprotein structurally characterized by an N-terminal region containing an IgV-like and a mucin domain. In humans, KIM-1 is undetectable in healthy subjects but is strongly expressed and released by injured proximal tubular epithelial cells. These characteristics, along with the persistent expression in tubular cells until damage recovery, and the rapid cleavage of its ectodomain which can be detected in urine contribute to making KIM-1 an ideal biomarker of tubular injury [47].

Clinical studies have shown that urinary KIM-1 (uKIM-1) is higher in patients with ischemic renal injury compared to controls and also that it is a predictor for the risk of developing AKI [48–50]. Additional studies have documented that KIM-1 and its urinary derivative are upregulated in various kidney diseases including diabetic nephropathy, focal glomerulosclerosis, membranoproliferative glomerulonephritis, and IgA nephropathy [51]. Urinary KIM-1 also predicts graft loss in kidney transplant recipients [52–54] and this predictive role has an important role in the era of kidney transplants from expanded criteria donors [55, 56].

In recent years there has been a growing interest in tumor markers not only for diagnostic purposes but also to improve the predictive power of clinical and pathological factors in prognostic models [57–59]. It has been shown that KIM-1, besides its utility as a biomarker of renal injury, could have a diagnostic role in renal cell carcinoma (RCC).

RCC accounts for about 3% of all adult malignancies and, even if many proteins have been investigated as potential biomarkers [60] in recent years, their diagnostic and prognostic relevance is still under debate. In this scenario, Han et al. have demonstrated the expression of KIM-1 in RCC tissue samples. Moreover, uKIM-1 was detectable in the urine of RCC patients before nephrectomy but showed markedly reduced levels after surgery [61].

Considering the characteristics of uKIM-1, which contribute to its utility as an early and sensitive biomarker for kidney injury, its role was recently analyzed in the setting of obstructive nephropathy. In a case-control prospective study performed in children with severe hydronephrosis due to UPJO, Wasilewska et al. showed that, like uNGAL, uKIM-1 concentrations were significantly higher in affected children compared to control groups [45]. Moreover, three months after surgery, uKIM-1 values had decreased significantly even if they were still higher than the concentrations found in children with dilated not obstructed kidneys. A more recent study investigated the diagnostic performance of uKIM-1 and uNGAL for AKI in 90 patients with obstructive nephropathy [62]. Both uKIM-1 and uNGAL concentrations were higher in AKI patients than non-AKI patients, and the uKIM-1 value measured 72 hours after surgery was an independent predictor of renal outcome in patients with AKI.

## 5. Conclusions

In the last decades much has been learned about the pathophysiology of obstructive nephropathy. This better knowledge has led to the discovery of novel biomarkers for diagnostic and prognostic purposes. We have learned that obstructive nephropathy is an evolving disease in which the renal damage continues even after relief of the obstruction. In particular, it has been demonstrated that the time of relief is the most important factor in predicting long-term renal function deterioration. Patients who underwent late surgical relief of an obstruction suffer from decreased renal function in the long term, as a consequence of the molecular events triggered by previous acute injury, which lead to progressive interstitial fibrosis and tubular apoptosis.

In this setting, the EGF/MCP-1 ratio, uNGAL, and uKIM-1 are useful early biomarkers of progressive renal damage and could have a potential role in predicting the long-term renal outcome. Additional comprehensive validation studies are warranted to confirm the utility of these biomarkers in clinical practice.
